# Pituitary hormone level changes and hypxonatremia in aneurysmal subarachnoid hemorrhage

**DOI:** 10.3892/etm.2013.1068

**Published:** 2013-04-18

**Authors:** AISHA MAIMAITILI, MIJITI MAIMAITILI, AIKEREMU REXIDAN, JUNYI LU, KUERBAN AJIMU, XIAOJIANG CHENG, KUN LUO, DUISHANBAI SAILIKE, YUAN LIU, KADEER KAHEERMAN, CHANGJIU TANG, TINGRONG ZHANG

**Affiliations:** Department of Neurosurgery, The First Affiliated Hospital of Xinjiang Medical University, Urumqi, Xinjiang 830054, P.R. China

**Keywords:** aneurysm, subarachnoid hemorrhage, hyponatremia, pituitary hormone

## Abstract

The aim of this study was to investigate the changes in serum pituitary hormone levels and the mechanism of hyponatremia in aneurysmal subarachnoid hemorrhage (SAH). Nuclear medical tests and serum electrolyte monitoring were performed in 49 aneurysmal SAH cases and 10 healthy volunteers. The levels of serum pituitary hormones were significantly higher in the SAH patients compared with the control group on days 1–3 and 7–9 after SAH onset (P<0.05). The peak value occurred on days 7–9. The rate of hyponatremia was 49.0% in the 49 SAH patients. The incidence of severe hyponatremia was significantly higher in Fisher grades III–IV and Hunt-Hess grades III–IV compared with Fisher grades I–II and Hunt-Hess grades I–II, respectively (P<0.05). There was no correlation between the site of aneurysm and the rate of hyponatremia. The incidence of symptomatic cerebral vasospasm was significantly higher in the hyponatremia group and Fisher grades III–IV compared with the normal serum sodium group and Fisher grades I–II, respectively. Serum pituitary hormone levels were positively correlated with blood loss and disease severity in patients with aneurysmal SAH. Hyponatremia may be considered an important indicator of SAH. SAH patients are likely to benefit from intense monitoring and regulation of serum sodium.

## Introduction

Subarachnoid hemorrhage (SAH) is a common term for the sudden rupture of blood vessels of the brain and flow of blood into the subarachnoid cavity, which occurs due to a variety of reasons. Spontaneous SAH accounts for ∼15% of cases, an incidence second only to hypertensive cerebral hemorrhage, and cerebral infarction ranks third (1). Following rupture of the aneurysm, the blood flows into the subarachnoid cavity and degradation products are released due to the rupture of red blood cells, resulting in the local release of vasoactive substances, causing spasm of Willis’ arterial circle at the brain skull base. The rupture of an aneurysm at the anterior communicating artery may cause spasm of the anterior communicating artery and perforating artery. The perforating artery of the anterior communicating artery supplies blood to the mouth of the hypothalamus; therefore, when spasm occurs to this artery, hypothalamic ischemic damage is caused, resulting in hormone secretion abnormalities and hyponatremia.

Hyponatremia is one of the most common complications in intracranial aneurysmal SAH. In related literature, the incidence is ∼30–40% and once hyponatremia occurs, the fatality rate increases by 14.3% (2,3). Currently, it is considered that the occurrence of hyponatremia secondary to SAH is caused by the following: syndrome of inappropriate antidiuretic hormone (SIADH) and cerebral salt wasting syndrome (CSWS). SIADH is a syndrome in which excessive secretion of antidiuretic hormone (ADH) is caused by stimulation of the hypothalamus with various traumatic factors, resulting in the enhancement of water re-absorption in the kidney collection tube and the distal convoluted tubule, causing fluid retention and dilutional hyponatremia. Although the secretion of ADH in the plasma of CSWS patients is normal, it is always accompanied by urinary sodium excretion, as well as reductions of extracellular fluid and circulating blood volume, causing hyponatremia. In 2009, a multidisciplinary working group in Florida University, USA, developed diagnostic criteria for SIADH and CSWS by reviewing a large number of studies using evidence-based medicine (4). The clinical manifestations and laboratory test results of the two diseases are similar; however, the pathogenesis and, thus, the treatment methods differ. Therefore, differentiating between the two syndromes is of great significance for the treatment. It is important to explore whether the incidence of hyponatremia in SAH patients is associated with the amount of bleeding and severity of the condition and whether hyponatremia may be used as an indicator of severity and poor prognosis.

The present study aimed to observe the indicators adrenocorticotropic hormone (ACTH), thyroid-stimulating hormone (TSH), follicle-stimulating hormone (FSH), luteinizing hormone (LH), prolactin (PRL) and growth hormone (GH) levels in aneurysmal SAH patients in the acute phase, using Fisher computed tomography (CT) classification for the amount of bleeding and Hunt-Hess grade for the severity of the condition. By dynamic observation of these indicators, we aimed to determine the correlation between the various indicators and the amount of bleeding and staging of the disease, to investigate whether the changes in pituitary hormone function serve as effective indicators for judging the condition and guiding clinical treatment.

## Subjects and methods

### Subjects

The clinical data from 49 patients with intracranial aneurysms admitted to the Department of Neurosurgery of the First Affiliated Hospital of Xinjiang Medical University (Urumqi, China) from March 2011 to November 2011 were selected. Of the 49 patients, 22 were male and 27 were female, with a male to female ratio of 1:1.2. The age of the patients ranged from 28 to 78 years, with a mean age of 53.14±12.75 years. All patients received head computed tomography angiography (CTA) examination and diagnosis was confirmed by surgery; all cases received surgical treatment within 48 h. Nineteen patients had anterior cerebral artery and anterior communicating artery aneurysm, 18 patients had carotid-posterior communicating artery aneurysm and 12 patients had cerebral artery aneurysm. The severity was classified in accordance with the Hunt-Hess grade (5) and the amount of bleeding was classified in accordance with the modified Fisher classification (6) (Table I). The control group included 10 healthy individuals without endocrine diseases; these were 4 males and 6 females, aged 29–58 years with a mean age of 47.9±8.69 years.

The study was conducted in accordance with the Declaration of Helsinki and with approval from the Ethics Committee of the First Affiliated Hospital of Xinjiang Medical University. Written informed consent was obtained from all participants.

### Diagnostic criteria of hyponatremia

Medical system diseases and hyponatremia derived from long-term salt restriction and severe vomiting were first excluded by assesssing the medical history, X-ray examination, abdominal B ultrasound and chest and abdomen CT examination. In the clinical research process, hyponatremia was defined as a serum sodium level <135 mmol/l lasting for at least one day; a serum sodium level in the range of 125 to 134 mmol/l was defined as mild hyponatremia and a serum sodium level of <125 mmol/l was defined as severe hyponatremia.

### Diagnostic criteria of symptomatic cerebral vasospasm (SCVS)

The diagnostic criteria of SCVS were as follows: i) within 4 to 9 days after SAH, progressive increase of headache with continuous and unrelieved symptoms, blood pressure increase and deterioration of consciousness occurred, and the location of the affected area of the nervous system was consistent with the perfusion area of the impaired cerebral artery; ii) rebleeding ruled out by brain CT scan; and iii) imaging demonstrating an infarct area in the corresponding dominated area or digital subtraction angiography (DSA) examination revealing artery stenosis or occlusion.

### Surgical method and postoperative treatment

After the diagnosis of the 49 patients was confirmed, the aneurysms of 39 cases were surgically clipped under a craniotomy microscope and 10 patients received femoral artery puncture interventional embolization. Following surgery, in addition to conventional therapy, the 3H program expansion treatment was administered for therapy and Nimotop was administered for the prevention of cerebral vasospasm (CVS). When the meningeal irritation was heavier, lumbar puncture or continuous lumbar drainage was conducted to release bloody cerebrospinal fluid (BCSF), so as to relieve headaches and CVS.

### Methods

Following admission, 3 ml peripheral venous blood was collected from all patients during the same time period in the early morning 1–3 days after the onset (non-menstrual blood was drawn from females) and sent for testing in the Department of Nuclear Medicine of our hospital. Blood was drawn again from the 14 dynamic observation patients from 7–9 days and 13–15 days after the onset of symptoms and sent to the Department of Nuclear Medicine for testing. Following admission, serum was simultaneously collected to analyze electrolytes and was reviewed several times following surgery to determine the changes in sodium ion concentration during the course of the disease. Venous blood was drawn from the control group after 30 min of repose and sent to the Department of Nuclear Medicine for testing.

### Statistical analysis

Statistical analysis was performed using SPSS 19.0 software (SPSS Inc., Chicago, IL, USA). All data are expressed as mean ± standard deviation and all data were tested with the normality test, suggesting that the results were normally distributed (P>0.05). The homogeneity test of variance was performed among the groups, suggesting homogeneity of variance (P>0.05) and single factor analysis of variance was adopted for the comparison of the data among the three groups. The Chi-square test was applied for the analysis of hyponatremia count data and α=0.05 was selected as the test level of all tests.

## Results

### Correlation between Hunt-Hess grade and blood serum indicators

As shown in Table II, compared with the indicators of the control group, the indicators of patients of Hunt-Hess grades I and II were not statistically significantly different (P>0.05) but those of patients of Hunt-Hess grades III and IV–V were statistically significantly different (P<0.05). The indicators of patients of grades III and IV–V were significantly different (P<0.05) from those of patients of grades I and II, and the indicators of patients of grade IV–V were significantly different from those of patients of grade III (P<0.05).

### Correlation between Fisher grade and blood serum indicators

As shown in Table III, the indicators of patients of Fisher grades I and II were not significantly different from those of the control group (P>0.05); however, the differences in the indicators of patients of Fisher grades III and IV–V from those of the control group were statistically significant (P<0.05). When the indicators of patients of grades III and IV–V were compared with those of patients of grades I and II, the differences were statistically significant (P<0.05) and when the indicators of patients of grades IV–V were compared with those of patients of grade III, the differences were also statistically significant (P<0.05).

### Correlation between the disease stages of the 14 dynamic observation cases and blood serum indicators

As shown in Table IV, the indicators were significantly increased, compared with those of the control group, at 1–3 days and 7–9 days after the onset of the disease (P<0.05). When the indicators at 13–15 days were compared with those of the control group, the differences were not statistically significant (P>0.05). During the whole disease period, the increase of the mean value of the indicators at 7–9 days was the most evident and when compared with that at 1–3 days and 13–15 days, the difference was significant (P<0.01).

### Correlation between mild and severe hyponatremia and Fisher grade

As shown in Table V, among the 27 patients of Fisher grade III–IV, 9 patients had severe hyponatremia; while among the 22 patients of Fisher grade I–II, 2 patients presented severe hyponatremia and the difference between the two groups was statistically significant (P<0.05).

The correlation between mild and severe hyponatremia and Hunt-Hess grade is shown in Table VI. Of the 25 patients of Hunt-Hess grade III–V, there were 9 cases of severe hyponatremia and of the 24 patients of Hunt-Hess grade I–II, there were 2 cases of severe hyponatremia; the difference was statistically significant (P<0.05).

### Incidence of hyponatremia for different sites of aneurysm

As shown in Table VII, using the nonparametric rank-sum test, when the proportions of mild hyponatremia and severe hyponatremia in the anterior cerebral-anterior communicating artery aneurysm group was compared with those of the internal carotid-posterior communicating artery aneurysm and carotid cerebral artery aneurysm groups, all P-values were >0.05, indicating that the sites of aneurysm demonstrated no significant correlation with the proportion of hyponatremia.

### Correlation between hyponatremia and SCVS

As shown in Table VIII, 5 of the 25 cases in the normal serum sodium group and 14 of the 24 cases in the hyponatremia group presented SCVS; the difference between the two groups was significant (P<0.05), indicating hypernatremia secondary to aneurysmal SAH is closely correlated with SCVS.

### Correlation between Fisher grade and SCVS

As shown in Table IX, among the 27 patients of Fisher grade III–IV, 15 cases presented SCVS; while among the 22 patients of Fisher grade I–II, 4 cases presented SCVS and the difference between the two groups was statistically significant (P<0.05). The results indicate that the Fisher grade is closely correlated with SCVS, indicating that the occurrence and extent of SCVS correlates with the severity of SAH.

## Discussion

Based on the analysis of the correlation between serum indicators and the amount of bleeding and severity of SAH, we consider that the increases in ACTH, FSH, LH, PRL, GH and TSH levels are positively correlated with the amount of bleeding and the severity of the disease. In 2006, Wang *et al* (7) identified in a study of the changes in hypothalamus-pituitary hormones in patients with intracranial aneurysm rupture that with increased bleeding and increased Hunt-Hess grade, the changes in the indicators were more evident. Zhou *et al* (8) identified in a study of the concentration of pituitary hormones in the cerebrospinal fluid (CSF) of patients with aneurysmal SAH that changes in pituitary hormone levels in the CSF of patients with aneurysmal SAH is an indicator of the degree of CVS and the prognosis of patients.

The results reported in this study are similar to those of previous studies. The hypothalamus-pituitary dysfunction mechanism is not fully understood; however, its possible mechanism may be as follows: i) spasm of Willis’ arterial circle, cerebral blood circulation disorder and disruption of pituitary portal system circulation caused by direct stimulation of bloody fluid following hemorrhage and products derived from erythrocyte lysis, leading to ischemic injury of the pituitary; ii) direct compression of a blood clot leads to displacement and destruction of the brain tissue structure, affecting the function of the hypothalamus; iii) formation of a hematoma around the Willis’ circle under oppression spreads to the hypothalamus and pituitary stalk; iv) blood is directly ruptured into the ventricular system and the hypothalamus is directly invaded, leading to the release of increased levels of gonadotropin-releasing hormone (GRH), thyrotropin-releasing hormone (TRH) and corticotropin-releasing hormone (CRH), to promote the increased secretion of pituitary ACTH, FSH, LH, PRL, GH and TSH (2). If these indicators are significantly increased 1–3 days after the onset of the lesion, often indicating that the illness of the patient is severe and the amount of bleeding is high, the patient’s condition should be considered serious and appropriate measures should be taken as soon as possible. Therefore, the results indicate that changes in serum indicators reflect the amount of bleeding and severity of the illness to a certain extent.

By dynamic observation of the pituitary hormones of 14 patients, we identified that, 7–9 days after the onset of the disease, the indicators were significantly increased, as compared with those at 1–3 days and 13–15 days. The possible mechanism may include: i) the stress response following bleeding leads to increased secretion of pituitary hormones (9); ii) direct damage of the hypothalamus and anterior pituitary lead to the direct release of hormones into the blood; iii) the aggravation of the intracerebral edema following hemorrhage affects hypothalamic function (10); and iv) incidence of CVS is in the peak period within 7–9 days of the onset of the disease; therefore, ischemic infarction or hemorrhage is likely to occur affecting hypothalamic-pituitary function (11). Therefore, in clinical practice, these indicators are likely to progressively increase 7–9 days after the onset of the disease. Great attention should be paid to the occurrence of CVS and early anti-CVS treatment should be administered to prevent progressive aggravation of the patient’s condition. Through this study, we have gained an improved understanding of the dynamic evolution of aneurysmal SAH, which is likely to help in evaluating the condition, particularly the occurrence of CVS, to determine the appropriate treatment and to estimate prognosis.

According to the study by Sherlock *et al* (2), 41.3% of hyponatremia secondary to aneurysmal SAH occurs 1–3 days after hemorrhage and 31.8% occurs after 7 days. Its clinical manifestations are dependent on the extent of hyponatremia and its progress rate; during the treatment of hyponatremia, correcting too quickly or slowly causes osmotic demyelination syndrome (ODS) and even mortality (12–14). Therefore, the correct approach must be taken for the treatment of hyponatremia secondary to aneurysmal SAH, otherwise the prognosis of patients is likely to be affected. We assessed the clinical data of 49 patients with aneurysmal SAH to investigate its incidence, clinical features and pathogenesis. Of the 49 patients with aneurysmal SAH, 24 patients had hyponatremia, an occurrence rate of 49.0% (24/49). In addition, comparative analysis was conducted by grouping and grading and the results are reported as follows: i) the incidence of severe hyponatremia of Fisher grades III–IV was significantly higher than that of Fisher grades I–II; ii) the incidence of severe hyponatremia of Hunt-Hess grades III–IV was significantly higher than that of Hunt-Hess grades I–II; iii) there was no significant correlation between the site of aneurysm and hyponatremia; iv) the incidence of SCVS in the hyponatremia group was significantly higher than that of the normal serum sodium group; and v) the incidence of SCVS of Fisher grades III–IV was significantly higher than that of Fisher grades I–II.

Harrigan (15) considered that, within 2 weeks of brain injury, hyponatremia is often caused by CSWS, with an incidence rate higher than that of SIADH and the reason is that, at the onset of hyponatremia, a large amount of sodium is discharged from urine, resulting in hypovolemia and high urinary sodium, while the ADH levels in the blood are normal. Kurokawa *et al* (16) considered that the main cause of hyponatremia is CSWS, while natriuretic peptide (NP) is the main cause of CSWS. This view has been gradually accepted. The present study confirms that, in the occurrence and development process of CSWS, NP plays an important role.

Ogawasara *et al* (17) stated that differentiation of SIADH and CSWS may be confirmed by monitoring weight and central venous pressure. When water is discharged from the body of a patient with CSWS, a large amount of urine sodium is also discharged, resulting in a serious decline in blood volume and decrease in central venous pressure; SIADH is manifested as fluid retention, increased blood volume and increased central venous pressure. Therefore, evaluation of the volume status of patients and measurement of central venous pressure, lung capillary vessel wedge and plasma volume are usually adopted. Among the 49 cases in this study, 24 cases presented hyponatremia, three of which were diagnosed as SIADH. These patients often presented no dehydration; however, SIADH manifested as increased blood volume, decreased plasma osmolality and a hematocrit level lower than the normal value. Twenty-one cases were diagnosed as CSWS. These patients presented dehydration, elevated heart rate, dry skin, eye socket sinking, reduced urine output and dark urine, and a hematocrit level higher than the normal value.

The majority of studies have identified that the occurrence of aneurysmal SAH hyponatremia is closely correlated with CVS. CTA or DSA conducted within 4–12 days of hemorrhage revealed a high incidence of CVS (∼30–70%) (18–20), and ∼30% of the patients with CVS presented clinical symptoms (SCVS). Nineteen of the 49 cases of aneurysmal SAH in the present study had SCVS, an incidence of 38.8%, consistent with results reported in the previous studies.

A study (21) identified that proteins, amines and other substances are involved in the occurrence of CVS. The common pathway of these substances is to promote an influx of calcium in vascular smooth muscle cells, as well as calcium release from intracellular calcium stores, which causes a cytosolic calcium overload and is the basis for the application of calcium antagonists in the treatment of CVS.

A study by Shi *et al* (22) suggested that the abnormal blood clotting mechanism of patients is also a cause of SCVS. Following SAH, the coagulation-fibrinolysis system is activated and plasma is in a hypercoagulable state, prone to the formation of tiny blood clots that lead to delayed ischemic neurological deficits (DIND). However, following SAH, the use of large amounts of dehydrating agents and reductions in extracellular fluid volume resulting from CSWS, blood concentration, increased blood viscosity and aggravation of cerebral edema due to hyponatremia affect the brain micro-circulation, which further aggravates the cerebral ischemia and leads to corresponding clinical symptoms. Therefore, it is necessary to attend to the treatment of SCVS secondary to aneurysmal SAH. Of the 49 patients with aneurysmal SAH in this study, the incidence rate of SCVS of Fisher grades III–IV was 55.6% (15/27) and the incidence rate of SCVS of Fisher grades I–II was 18.2% (4/22); the difference between the rates was statistically significant (P<0.05). The results indicate that Fisher grade is closely correlated with SCVS, demonstrating that the occurrence and extent of SCVS correlates with the severity of SAH. The incidence of SCVS in the hyponatremia group was 58.3% (14/24) and in the normal serum sodium group was 20.0% (5/25); the difference between the two groups was significant (P<0.05), demonstrating that hyponatremia secondary to aneurysmal SAH is closely correlated with SCVS. The results indicate that hyponatremia correlates with the occurrence of SCVS, which may be used as an indicator to determine the prognosis of patients with aneurysmal SAH.

For the treatment of CVS combined with aneurysmal SAH, the 3H treatment program (hypertension, hypervolemia and hemodilution) is adopted in the majority of cases. This treatment reduces blood viscosity, increases cerebral perfusion pressure, improves cerebral oxygen supply and the cerebral insufficiency due to CVS secondary to SAH, and prevents the occurrence of DIND. Due to the administration of volume expansion therapy, there is a risk of increased intracranial pressure, thus ECG monitoring should be performed to monitor changes in vital signs. Youmans Neurological Surgery (23) states that nimodipine is the first choice drug for the management of CVS in SAH and should be continued for 21 days. The meta-analysis by the Cochrane Centre in 2007 (24) revealed that nimodipine improves ischemic changes secondary to aneurysmal SAH, resulting in a decline in the fatality rate and disability of CVS. Treatment should be initiated as early as possible, using sufficient and safe quantities and ensuring that the entire process is completed The starting dose is generally 0.5 mg/h and if there are no significant changes in blood pressure, the dosage may be increased to 1 mg/h after 2 h. The 24-hour intravenous dose is 24–48 mg; as the half-life of nimodipine is 1.5 h, continuous infusion with an intravenous infusion pump is suggested. Nineteen cases of SCVS were treated with 3H treatment. Based on the adequate volume expansion therapy, Nimotop was administered at a pump flow rate of 3 ml/h for 24 h and ECG was performed to monitor changes in vital signs. After continuous use for ∼10 days, oral tablets at a dosage of 60 mg/4 h were administered. A good efficacy was achieved and of the 19 patients, 12 survived during the CVS peak period, 3 succumbed and 4 abandoned the treatment due to economic reasons and were automatically discharged. Therefore, the key link in reducing the occurrence of CVS and improving the prognosis is early diagnosis and early treatment.

In summary, changes in ACTH, FSH, LH, PRL, GH and TSH levels in the serum of aneurysmal SAH patients are positively correlated with the amount of bleeding and severity of the condition. Dynamic observation revealed that an increase in the indicators within 7–9 days was most evident, which, to a certain extent, reflects the extent of damage to the hypothalamus-pituitary system and may be used as an objective indicator to determine the changes in the condition of patients and to guide clinical treatment. Hyponatremia is a common complication of aneurysmal SAH and it is an important indicator reflecting the severity and prognosis of aneurysmal SAH. Improved understanding of the mechanism of hyponatremia complicated with aneurysmal SAH in patients is the key for effective treatment.

## Figures and Tables

**Table I:** Hunt-Hess and Fisher grade of clinical cases.

Grade	Hunt-Hess grade				Fisher grade			
	
	I	II	III	IV–V	I	II	III	IV
N	9	15	13	12	8	14	16	11

**Table II:** Correlation between Hunt-Hess grade and serum ACTH, FSH, LH, PRL, GH and TSH levels.

Group	N	ACTH (pg/ml)	FSH (mIU/ml)	LH (mIU/ml)	PRL (ng/ml)	GH (ng/ml)	TSH (*μ*IU/ml)
Control	10	40.17±13.94	4.37±1.89	3.45±1.45	6.43±2.19	1.28±0.78	3.12±0.98
Grade I	9	47.87±14.66	5.44±2.14	3.78±1.84	6.87±1.98	1.59±0.93	3.68±1.37
Grade II	15	76.81±35.67	7.07±2.68	6.16±2.76	9.42±4.15	2.83±0.95	5.69±2.06
Grade III	13	115.31±36.24	11.62±3.45	10.97±4.36	16.68±4.67	5.03±2.32	7.94±3.08
Grade IV–V	12	155.17±33.28	16.22±4.21	14.60±3.68	18.82±2.56	8.02±1.02	12.44±2.92

Data are presented as mean ± standard deviation. ACTH, adrenocorticotropic hormone; FSH, follicle-stimulating hormone; LH, luteinizing hormone; PRL, prolactin; GH, growth hormone; TSH, thyroid-stimulating hormone.

**Table III:** Correlation between Fisher grade and serum ACTH, FSH, LH, PRL, GH and TSH levels.

Group	N	ACTH (pg/ml)	FSH (mIU/ml)	LH (mIU/ml)	PRL (ng/ml)	GH (ng/ml)	TSH (*μ*IU/ml)
Control	10	40.17±13.94	4.37±1.89	3.45±1.45	6.43±2.19	1.28±0.78	3.12±0.98
Grade I	8	45.32±13.38	5.67±2.17	3.74±1.96	6.91±2.11	1.68±1.01	3.92±1.25
Grade II	14	68.26±29.82	6.66±2.87	5.46±2.73	8.36±3.98	2.58±1.14	5.21±2.52
Grade III	16	126.65±37.22	10.64±3.06	10.15±2.99	15.94±3.05	4.50±1.58	8.12±2.83
Grade IV	11	145.37±37.33	17.45±2.89	15.97±3.09	19.81±3.06	8.62±1.41	12.42±3.09

Data are presented as mean ± standard deviation. ACTH, adrenocorticotropic hormone; FSH, follicle-stimulating hormone; LH, luteinizing hormone; PRL, prolactin; GH, growth hormone; TSH, thyroid-stimulating hormone.

**Table IV:** Dynamic observation of the correlation between the illness period and serum ACTH, FSH, LH, PRL, GH and TSH levels in 14 cases.

Group	ACTH (pg/ml)	FSH (mIU/ml)	LH (mIU/ml)	PRL (ng/ml)	GH (ng/ml)	TSH (*μ*IU/ml)
Control	40.17±13.94	4.37±1.89	3.45±1.45	6.43±2.19	1.28±0.78	3.12±0.98
After illness (days)						
1–3	64.15±29.42	7.10±2.85	4.93±3.01	8.95±3.60	2.18±1.12	4.61±1.75
7–9	146.88±42.53	12.95±4.53	9.24±4.48	21.75±4.94	4.03±1.31	7.29±1.83
13–15	38.34±20.70	6.74±4.12	3.69±3.00	6.95±3.00	1.37±0.77	2.91±1.75

Data are presented as mean ± standard deviation. ACTH, adrenocorticotropic hormone; FSH, follicle-stimulating hormone; LH, luteinizing hormone; PRL, prolactin; GH, growth hormone; TSH, thyroid-stimulating hormone.

**Table V:** Correlation between mild hyponatremia, severe hyponatremia and Fisher grade.

Group	N	Mild hyponatremia	Severe hyponatremia
Grade I–II	22	4 (18.1%)	2 (9.0%)
Grade III–IV	27	9 (33.3%)	9 (33.3%)

**Table VI:** Correlation between mild hyponatremia, severe hyponatremia and Hunt-Hess grade.

Group	N	Mild hyponatremia	Severe hyponatremia
Grade I–II	24	6 (25.0%)	2 (8.3%)
Grade III–IV	25	7 (28.0%)	9 (36.0%)

**Table VII:** Incidence of mild hyponatremia for different sites of aneurysm.

Site of aneurysm	N	Mild hyponatremia	Severe hyponatremia
Anterior cerebral or communicating artery	19	6 (31.6%)	6 (31.5%)
Carotid-posterior communicating artery	18	5 (27.8%)	3 (16.7%)
Cerebral artery	12	2 (16.7%)	2 (16.7%)

**Table VIII:** Correlation between mild hyponatremia and symptomatic cerebral vasospasm (SCVS).

Group	N	SCVS	Incidence
Normal blood sodium	25	5	20%
Low blood sodium	24	14	58.3%

**Table IX:** Correlation between Fisher grade and symptomatic cerebral vasospasm (SCVS).

Group	N	SCVS	Incidence
Grade I–II	22	4	18.2%
Grade III–IV	27	15	55.6%
